# Theoretical Studies of Non-Metal Endohedral Fullerenes

**DOI:** 10.3390/nano15161287

**Published:** 2025-08-21

**Authors:** Zdeněk Slanina, Filip Uhlík, Takeshi Akasaka, Xing Lu, Ludwik Adamowicz

**Affiliations:** 1 Department of Chemistry and Biochemistry, University of Arizona, Tucson, AZ 85721, USA; ludwik@arizona.edu; 2 State Key Laboratory of Materials Processing and Die & Mould Technology, School of Material Science and Engineering, Huazhong University of Science and Technology, Wuhan 430074, China; akasaka@tara.tsukuba.ac.jp (T.A.); lux@hust.edu.cn (X.L.); 3 Department of Physical and Macromolecular Chemistry, Faculty of Science, Charles University, Albertov 6, 128 43 Praha 2, Czech Republic; filip.uhlik@natur.cuni.cz

**Keywords:** fullerene non-metal endohedrals, encaged small molecules, stability evaluations, encapsulation energy and Gibbs energy, quantum-chemical calculations

## Abstract

This article presents computational studies of non-metal fullerene endohedrals, which are useful for understanding and interpreting experimental results. The encapsulated non-metal species are simple molecules like H_2_, N_2_, CO, HF, NH_3_, H_2_O_2_, H_2_O, and their aggregates. Predictions of thermodynamic stability and reaction populations are reviewed, based on quantum-chemical and statistical–thermodynamic treatments. As fullerene syntheses are performed at high temperatures, some of the calculations are based on both the encapsulation potential energy and the encapsulation Gibbs energy changes.

## 1. Introduction

Recently, endohedral encapsulation in fullerenes has been extended from metals to non-metal species (Table 1 presents illustrative examples). Metallofullerenes are produced by internal charge transfer. However, the charge transfer is not important for encapsulations of non-metal species—their formation is related [1] to weak interactions. N_2_@C_60_ and N_2_@C_70_ are examples of such non-metal endohedral fullerenes, produced [2] by means of high-temperature and high-pressure treatment. In fact, among two thousand C_60_ molecules, approximately one incorporates N_2_ [2]. Endohedrals with a nitrogen molecule can survive even several hours of heating at higher temperatures. The N_2_@C_60_ species is also observed [3] in the chromatographic analysis of nitrogen implantation in C_60_, otherwise primarily yielding N@C_60_ [4,5,6,7,8,9]. Endohedral fullerenes containing noble atoms [10,11,12,13,14] are also produced [10] by means of high-temperature and high-pressure techniques with a catalyst [13]. A more recent synthetic method for the encapsulation of non-metallic species (for example, hydrogen molecules [15] or water molecules [16]) put the molecule first in an open carbon cage and then its window was closed [17,18] by synthetic means. For example, complex synthesis produced [19] (H_2_O)_2_@C_70_. Carbon monoxide [20,21] or H_2_O_2_ [22,23] were also encapsulated in open-cage C_60_ derivatives. Obviously, such new fullerene encapsulations have also been calculated [24,25,26,27,28,29,30,31,32,33,34,35,36,37,38,39,40,41,42,43,44,45]—such theoretical treatments are surveyed in this report and illustrated with topical systems.

## 2. H_2_ Encapsulation in C_60_

Originally, non-metallic fullerene encapsulates were calculated primarily [30] with the traditional B3LYP functional and later on using other more recent DFT functionals [47,48,49,50] (the 6–31G*, 6–31G**, and other standard atomic basis sets). The calculations can be augmented with the evaluation of a correction known as a basis set superposition error (BSSE). The BSSE term is estimated using the counterpoise technique introduced by Boys and Bernardi [51]. BSSE correction ensures that all components of an association process are treated in the same basis set [52,53,54]. BSSE treatment was recently applied to various fullerenic species [55,56,57,58].

The DFT approach can be checked using a more advanced second-order Møller–Plesset perturbation treatment (MP2) [59]. There is also another more advanced technique—B2PLYP(D) treatment [60]—that combines the MP2 and DFT methods.

The MPWB1K and MP2 calculations [30] show a substantial encapsulation energy for H_2_@C_60_, i.e., the potential energy change during the reaction

(1) $$H_{2}\left(g\right)+C_{60}\left(g\right)=H_{2}@C_{60}\left(g\right).$$

The energy difference between the two methods is 4 kcal/mol (6–31G** basis, without the BSSE term). However, when moving to the still larger basis sets 6–311G(2d,2p) and (d,p)-6–311G**, the two methods differ by only 1 kcal/mol. Moreover, calculations with the SCS MP2 approach [61] reduce the energy gain by about 2 kcal/mol. Overall, it can be concluded [30] that the storage of H_2_ in the C_60_ cage is associated with a gain of about −4 kcal/mol in potential energy.

The influence of molecular structure optimizations for H_2_@C_60_ was also studied in [30]. It was found that, after full molecular geometry optimization was performed with the MPWB1K/6–31G** approach, the change in the energy reduction was only about 0.44 kcal/mol. The cage C-C bonds were changed by the encapsulation only at the level of the fourth digit. The cage 5/6-type C-C bonds optimized by the MPWB1K/6–31G** approach varied from 1.4377 Å to 1.4388 Å and the cage 6/6-type bonds varied between 1.3792 Å and 1.3798 Å. Let us note that orientation of the H_2_ unit towards two six-membered rings (and not towards two five-membered rings) brings about a change in the energy of just 0.1 kcal/mol. In other words, H_2_ can nearly freely rotate in the cage [62] (this feature also appears for some metallofullerenes [63]).

## 3. N_2_ Encapsulation in C_60_

The endohedral system was treated [26,28] with the B3LYP and PW91 functionals [64,65] in the 3–21G basis set. The lowest energy structure of N_2_@C_60_ exhibited orientation towards a pair of parallel five-membered rings; the endohedral belonged to the $D_{5d}$ point group of symmetry (denoted as a 5:5 structure [28]). The subsequent vibrational treatment confirmed that the N_2_@C_60_ 5:5 structure had no imaginary vibrational frequencies, i.e., it was indeed a local energy minimum. For N_2_@C_60_, the encapsulation energies defined by the process

(2) $$N_{2}\left(g\right)+C_{60}\left(g\right)=N_{2}@C_{60}\left(g\right).$$

had [26], at the MP2=FC/6–31G* level without and with BSSE correction (in the PW91/3–21G optimized geometry), substantial values of −17.5 and −9.28 kcal/mol, respectively, i.e., about two times higher potential energy gain compared to H_2_@C_60_.

The hypersurface of N_2_@C_60_ interaction energy is rather shallow. Consider a related N_2_@C_60_ stationary point that has its N_2_ molecule pointing towards a pair of parallel six-membered rings, denoted as 6:6 species. However, the 6:6 species is actually a saddle point (i.e., not a local energy minimum). Nevertheless, the 6:6 structure is still very close in energy to the 5:5 orientation—the calculations place the 6:6 structure less than 0.1 kcal/mol above the 5:5 species. This energy difference shows that the surface of interaction energy is flat. Consequently, encapsulated species should exhibit vibrational motion with large amplitudes at elevated temperatures. For the 5:5 N_2_@C_60_ optimized geometry, the shortest N-C distance calculated with the B3LYP/3–21G and PW91/3–21G approach is 3.046 and 3.049 Å, respectively. The optimized geometries themselves are not particularly sensitive to the selection of the DFT functional (in contrast to the encapsulation energetics). We also mention that the Mulliken atomic charge calculated on the nitrogen atoms is small—about 0.01 in elementary charge units—meaning that the charge transfer to the cage is negligible.

N_2_@C_60_ has 180 vibrational modes: one is the N-N bond stretching mode, five modes are vibrations of the N_2_ unit against the cage, and the remaining 174 modes are skeletal vibrations of the C_60_ cage. The N-N bond stretching frequency is only slightly affected by the encapsulation. Let us mention that the N-N stretching frequency increases in the B3LYP/3–21G method but decreases in the PW91/3–21G approach. The shifts in the N-N stretching frequency upon encapsulation are parallel with the calculated changes of the N-N bond length as in the B3LYP/3–21G approach, the bond is slightly shorter, while in the PW91/3–21G method, the N-N bond is longer by 0.0003 Å. The five frequencies for the N_2_-cage vibrations have rather low values, being similar to those of the La@C_82_ species [66].

**Figure 1:**
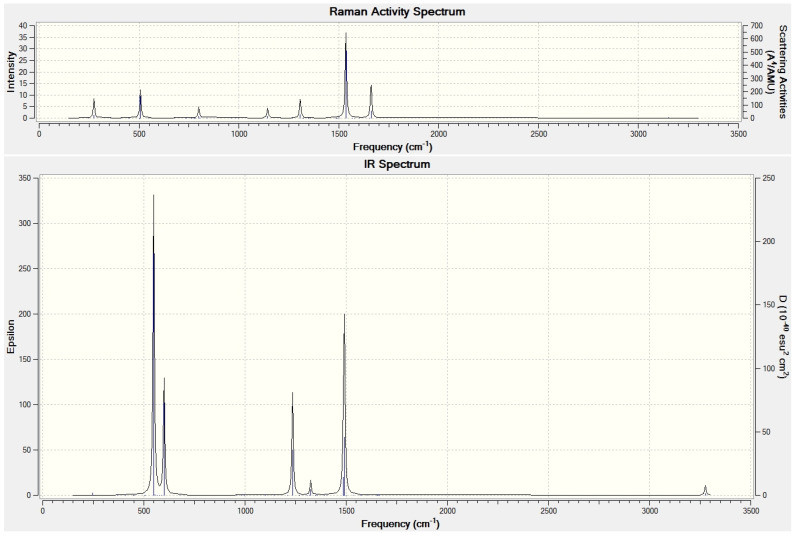
M06-2X/6–31++G** computed IR and Raman (top) spectrum [46] of CH_4_@C_60_.

The high icosahedral symmetry of C_60_ considerably reduces [67,68,69] its infrared (IR) and Raman spectra, e.g., just four $T_{1u}$ three-times degenerate vibrational species are actually present in its IR spectrum. However, when the icosahedral symmetry is reduced by encapsulation of N_2_, the spectral symmetry rules are, strictly speaking, different. Nevertheless, for N_2_@C_60_, just twelve cage modes exhibit substantial IR intensities. These vibrational modes active in the IR spectrum actually originate from the four $T_{1u}$ (three-times degenerate) modes of the pristine C_60_. Such degeneracy removal should be seen in the experimental vibrational spectra of N_2_@C_60_ once recorded. Similar features can, for example, be seen in the vibrational spectra [46] of CH_4_@C_60_ (Figure 1).

The encapsulation energy for Equation (2) is the energy change during the association process. The corresponding enthalpy term for a temperature *T*, $\Delta H_{T}^{o}$, is obtained by means of the ZPE (zero-point) vibrational energy and related heat-content terms. When the corresponding entropy term $\Delta S_{T}^{o}$ is calculated [70], we can arrive at the Gibbs energy change $\Delta G_{T}^{o}$ that describes the equilibrium thermodynamics. With the molecular partition functions based on the PW91/3–21G characteristics, the $T\Delta S_{T}^{o}$ contribution is equal to −5.95 kcal/mol at room temperature.

Let us note that the entropy evaluations need a careful approach to the rotational symmetry numbers, as some quantum-chemical program packages do not work with the proper symmetry number [69,71] (namely 60) of the icosahedral C_60_. Moreover, another important issue is the averaging effect of the encapsulate motions in the cage. If the encapsulate moves almost freely inside the C_60_ cage, it can recover the icosahedral symmetry for the endohedral. Thus, there are two ways [26,44,72,73] to reflect internal motions in the value of the endohedral symmetry number. One approach uses the static (non-icosahedral) symmetry of a rigid endohedral species, while the other considers the icosahedral symmetry produced by the fluctional behaviour [72,73] of the encapsulate.

When the entropy contribution $T\Delta S_{T}^{o}$ is used with the enthalpy $\Delta H_{T}^{o}$ term based on the MP2 = FC/6–31G* encapsulation energy corrected for the BSSE error, the final Gibbs energy change $\Delta G_{T}^{o}$ for reaction (2) at room temperature amounts [26] to −2.64 kcal/mol (the value corresponds to the term, for example, for the water dimerization in the gas phase [74]). The Gibbs energy term represents the driving thermodynamic force for the encapsulation process (2), described by the presently available computational data.

## 4. NH_3_ Encapsulation in C_60_

While N_2_@C_60_ is relatively well known based on experiments [2,3], a related endohedral NH_3_@C_60_ is yet to be prepared and has been characterized only by calculations [26,75,76], in particular by the energy gain for the encapsulation process:

(3) $${\mathrm{NH}}_{3}\left(g\right)+C_{60}\left(g\right)={\mathrm{NH}}_{3}@C_{60}\left(g\right).$$

The encapsulation energy calculated at the MP2 = FC/6–31G** level with the BSSE correction amounts [26] to −5.23 kcal/mol (i.e., about one half of the above energy gain with N_2_@C_60_, or comparable to the energy gain [77] in the N@C_60_ formation). Interestingly, the energy gain is slightly larger in the MP2 = FC/6–31G** approach (−17.89 kcal/mol without BSSE) than from the MP2 = FC/6–31G* method (−16.56 kcal/mol without BSSE). The influence of the DFT functional itself on the molecular structure optimization is again rather insignificant. For NH_3_@C_60_, calculations with the B3LYP/3–21G and PW91/3–21G geometries produce encapsulation energies of −5.23 and −5.25 kcal/mol, respectively. Calculations at room temperature for NH_3_@C_60_ yield an entropy term $T\Delta S_{T}^{o}$ of −5.46 kcal/mol. The Gibbs energy change $\Delta G_{T}^{o}$ for reaction (3) is [26] 1.53 kcal/mol, indicating lower stability of NH_3_@C_60_ compared to N_2_@C_60_.

Let us add for the sake of completeness that the $\Delta G_{T}^{o}$ changes (or equilibrium constants of encapsulation) should be considered [78] with the partial pressures of reaction components in experimental conditions. Moreover, catalytic [79,80] and kinetic [14,81,82,83] issues are also potentially involved.

## 5. CO Encapsulation in C_60_

CO@C_60_ was prepared [2,13] by heating under high pressure and placed [20,21] inside open-cage C_60_ derivatives. Evaluation [40] of the encapsulation process

(4) $$\mathrm{CO}\left(g\right)+C_{60}\left(g\right)=\mathrm{CO}@C_{60}\left(g\right)$$

for the 5:5 structure optimized at the MPWB1K/6–31G* level with the MP2 = FU/6–311 + G* approach including the BSSE correction gives an encapsulation energy of −12.5 kcal/mol. The thermodynamic treatment can produce for process (4) the encapsulation equilibrium constant $K_{p,enc,T}$, exhibiting a relatively fast temperature decrease. Nevertheless, under the synthetic conditions applied [2,13] for CO@C_60_ preparation (650 °C; 3000 atm), the relative CO@C_60_ fraction

(5) $$\frac{p_{CO@C_{60}}}{p_{C_{60}}}=p_{CO}K_{p,enc,T}$$

should be [40] about 3.5%. The evaluation yields an upper limit—the equilibrium should be enabled by a convenient kinetics that in the cage produces a temporary window [2,7,81,82,83,84,85]. In addition, a catalytic action can be required [13,81,85,86,87]. In comparison with the high-pressure and high-temperature encapsulations of water [36,37,38,42], the pressure of carbon monoxide is not controlled by its saturation regime (as the critical temperature of CO is [88] approximately 132 K), meaning that any pressure could in principle be considered. However, there should still be limitations on temperature in order not to allow, e.g., CO dissociation and the production of side derivatives, such as C_60_O [89,90,91]. An interesting issue concerning the smallest carbon cage [92,93] enabling the encapsulation of CO may also arise. This problem can be handled by means of topological techniques [94,95,96,97].

The IR spectrum of CO@C_60_ was simulated at the MPWB1K/3–21G level [40]. The stretching mode for encapsulated CO has a frequency of 2150 cm^−1^, which is 38 cm^−1^ smaller than at the same level calculated for gas-phase carbon monoxide. For CO encapsulated in derivatives of open C_60_ [20,21], vibrational frequencies were reported at 2125, 2118, and 2112 cm^−1^. These frequencies are smaller than the experimental fundamental of gas-phase CO [98] (2143 cm^−1^, shifting compared to the free CO by 18–31 cm^−1^). Thus, agreement between the observed and calculated frequency shifts is reasonable, considering differences in the treated terms (anharmonic vs. harmonic frequencies, open vs. closed cages).

Observed shifts in ^13^C NMR spectra are also reported [20,21] for CO placed in derivatives of open C_60_. ^13^C NMR shifts were evaluated with the MPWB1K/6–311 + G* method (in the MPWB1K/6–31G* calculated molecular structure). The experimental NMR shifts [20,21] are smaller by about 10 ppm than those observed for carbon monoxide in solution, while this reduction for the calculated shifts [40] is about 9.7 ppm.

The dipole moment for free CO [99] is rather small (0.122 D), and both experiments [99] and calculations [100,101,102] qualitatively conclude its charge distribution as C^−^O^+^, i.e., different from polarity derived [103,104] from electronegativity reasoning (however, the calculated term depends on the applied method). Interestingly, when CO@C_60_ is calculated with the MPWB1K/6–311 + G* approach, the charge on the oxygen atom is 0.92 and the charge on the carbon in CO is 0.28. The MPWB1K/6–311 + G*-calculated value of the endohedral dipole moment is only 0.04 D. The more advanced MP2 = FU/6–311 + G* method leads to a somewhat different picture—the charge on the O atom 1.55, that on the C atom is −1.02, and the system dipole moment is 0.128 D. Another issue is the change in polarizability after encapsulation [105,106], which can also be illustrated with the MPWB1K/3–21G method. For CO@C_60_, the isotropic polarizability is 62.33 Å^3^, for C_60_, it is 62.29 Å^3^, for CO, it is 1.22 Å^3^, and the relative reduction owing to encapsulation is equal to −1.18 Å^3^, in agreement with findings [106] for other fullerene-containing molecules.

## 6. H_2_O_2_ Encapsulation in C_60_

Recently, H_2_O_2_ was placed [22,23] inside a derivative with open-cage C_60_ at room temperature and atmospheric pressure. The observations were followed by model calculations [43] of the H_2_O_2_@C_60_ endohedral, describing the encapsulation

(6) $$H_{2}O_{2}\left(g\right)+C_{60}\left(g\right)=H_{2}O_{2}@C_{60}\left(g\right).$$

The M06-2X/6–31++G** molecular structure calculations give two isomers, A and B [43], among which the B isomer has a slightly higher energy, though only by 0.05 kcal/mol. The structural features of the encapsulated H_2_O_2_ molecule are not very different from those of free H_2_O_2_. The HOOH torsion angle is reduced by about 14° owing to the encapsulation. The shortest distances of the O and H atoms from the cage carbons are still in the range of non-bonding interactions. The calculated rotational constants of the H_2_O_2_@C_60_ isomers have basically identical values, meaning that the species would not be distinguished in rotational spectra.

The harmonic IR spectrum of H_2_O_2_@C_60_(A) was simulated [43] at the M06-2X/6–31++G** level. The free H_2_O_2_ possesses six normal modes of vibration; however, only four have substantial IR intensities in the M06-2X/6–31++G** evaluations, viz. 395 cm^−1^ (internal rotation or torsion mode [107]), 1339 cm^−1^ (bond-angle deformation), 3849 cm^−1^ (asymmetric O-H bond stretching), and 3849 cm^−1^ (symmetric O-H bond stretching). The vibrational modes exhibiting low IR intensity are 1038 cm^−1^ (O-O bond stretching) and 1478 cm^−1^ (bond-angle deformation). The four modes with higher IR intensity are basically also seen in H_2_O_2_@C_60_(A) (with some shifts in frequencies): 434 cm^−1^ (internal rotation or torsion), 1388 cm^−1^ (bond-angle deformation), 3806 cm^−1^ (asymmetric O-H bond stretching), and 3821 cm^−1^ (symmetric O-H bond stretching). The two H_2_O_2_ vibrational modes of lower intensity in the IR spectrum have endohedral frequencies of 1078 cm^−1^ (O-O bond stretching) and 1460.3 cm^−1^ (bond-angle deformation). Overall, the vibrational spectrum could help in H_2_O_2_@C_60_ laboratory identification once it is synthesized, and could even assist in the search for non-metallic fullerene endohedrals in the interstellar spectra (free hydrogen peroxide is in fact known to be present [108] in the interstellar space).

The charge distribution in hydrogen peroxide is not influenced significantly by encapsulation. In free H_2_O_2_, the M06-2X/3–21G-calculated charge on hydrogens is 0.387, while on oxygens, it is equal to −0.387. In H_2_O_2_@C_60_(A), the calculated charge on hydrogens reaches 0.411 on average, and on oxygens, the average is −0.381. Thus, there is quite a small negative charge transfer to the cage. The most negative charge on carbons is −0.0365 (with this particular carbon being located close to the hydrogen). On the other hand, the most positive charge on carbons reaches 0.0133 (this carbon is located close to the oxygen).

The potential energy gains in the formation of H_2_O_2_@C_60_(A) produced via process (6) are calculated [43] as −12.4 and −12.1 kcal/mol with the BSSE-corrected MP2 = FU/6–311++G** and B2PLYPD = FU/6–311++G** treatments, respectively. Thus, both of the considered advanced treatments, MP2 and B2PLYPD, produce almost identical results. The energy term differs by about 1 kcal/mol with the 6–311++G** and 6–31++G** standard basis sets (which agrees with the calculations [30] for H_2_@C_60_). The encapsulation energy is comparable to the values found [40] for CO@C_60_ as well as [26,28,30] for N_2_@C_60_. However, the gain in the potential energy is lower than that calculated [42] for (H_2_O)_2_@C_70_ (−18.4 kcal/mol) or for (H_2_O)_2_@$D_{2}$(22)-C_84_ (−17.4 kcal/mol).

Let us note that hydrogen peroxide has a critical temperature rather similar to that of water [88]. However, there are temperature restrictions to avoid hydrogen peroxide decomposition (and the formation of cage derivatives). Overall, a high-pressure and high-temperature preparation [2,10,11,12,13,14] of H_2_O_2_@C_60_ could in principle be possible.

## 7. Encapsulation of H_2_O and Its Aggregates into Fullerenes

**Figure 2:**
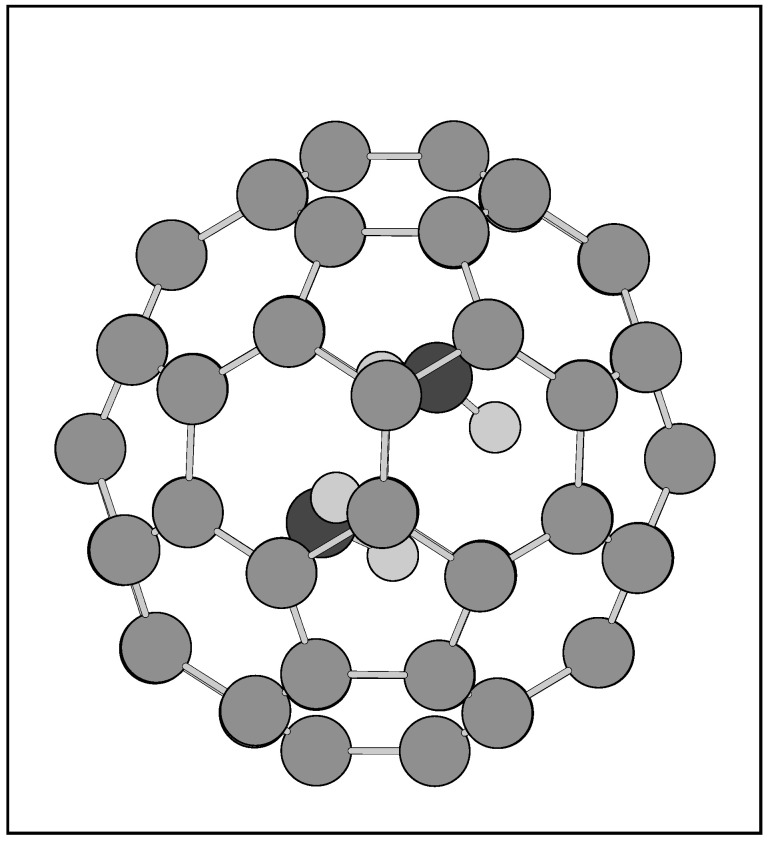
The M06-2X/6–31G** optimized structure [36] of (H_2_O)_2_@C_60_.

Compared to other non-metallic endohedral fullerenes like [2,13] N_2_@C_60_ or CO@C_60_, the water dimer would be rather large to fit in the C_60_ cage (though H_2_O@C_60_ can be prepared [18]). Figure 2 presents [36] the geometrical structure of (H_2_O)_2_@C_60_ optimized with the M06-2X/6–31G** method. The encapsulation energy of the water monomer calculated with the MP2/6–31G approach (without the BSSE term) is [27] −9.9 kcal/mol. However, the inclusion of the water dimer into C_60_ is repulsive at the same level, i.e., it exhibits [27] a positive (and not negative) encapsulation energy of 24.5 kcal/mol. The calculations instead indicated [36] C_84_ cages as convenient fullerenes for the water-dimer encapsulation, particular its two most stable species [109,110,111], conventionally labelled as $D_{2}$(22)-C_84_ and $D_{2d}$(23)-C_84_ (coded by their symmetries and serial enumeration numbers).

Beyond the previously discussed encapsulations, the C_84_ cages are represented [109,110,111,112,113,114,115,116,117,118,119] by twenty four [116] C_84_ isomers that obey the isolated pentagon rule (or IPR). However, the $D_{2}$(22)-C_84_ and $D_{2d}$(23)-C_84_ species represent the most populated isomers (though some minor species are reported by Dennis et al. [119]). Previously, based on their NMR observations, Kikuchi et al. [114] concluded that C_84_ has two major isomers, namely of $D_{2}$ and $D_{2d}$ symmetries observed in populations of 2:1. According to the semiempirical MNDO calculations [117], the $D_{2d}$ cage represents the lowest energy isomer (in other words, the global or lowest energy minimum), though the $D_{2d}$ species is placed just 0.5 kcal/mol below the $D_{2}$ cage. It should be noted that in the more sophisticated M06-2X/6–31G** approach [37], the $D_{2d}$ cage is placed only 0.3 kcal/mol below the $D_{2}$ species. In fact, the set of C_84_ isomers is the first reported case in which the observed most populated fullerene cage does not represent [72,120] the lowest energy isomer (such stability interchanges are also known for metallofullerenes [121,122,123,124,125]). C_84_ was calculated [109] as an isomeric set consisting of twenty four local energy minima—their energetics, molecular structures, and harmonic vibrational frequencies were produced by the semiempirical MNDO method [117]. The $D_{2d}$ isomer is the most populated species in the equilibrium mixture only up to a temperature of 276 K, while beyond this temperature, it is replaced by the $D_{2}$ isomer [109]. For example, at a temperature of 1000 K, the $D_{2}$(22)-C_84_ and $D_{2d}$(23)-C_84_ cage represents 60.3% and 34.2%, respectively, of the equilibrium 24-member isomeric mixture. In fact, the $D_{2}$ structure is chiral, which represents an important contribution [110] to the relative stability interchange. The two most populated C_84_ isomers can be isolated by chromatography [118] and belong to the most common higher fullerenes.

The calculations in [37] deal with a set of three gas-phase equilibrium processes:

(7) $$2H_{2}O\left(g\right)={\left(H_{2}O\right)}_{2}\left(g\right)$$

(8) $${\left(H_{2}O\right)}_{2}\left(g\right)+D_{2}\left(22\right)-C_{84}\left(g\right)={\left(H_{2}O\right)}_{2}@D_{2}\left(22\right)-C_{84}\left(g\right)$$

(9) $${\left(H_{2}O\right)}_{2}\left(g\right)+D_{2d}\left(23\right)-C_{84}\left(g\right)={\left(H_{2}O\right)}_{2}@D_{2d}\left(23\right)-C_{84}\left(g\right)$$

The water-dimerization enthalpy at a temperature of absolute zero $\Delta H_{0,2}^{o}$ calculated [37] by the G3&MP2 = (Full)/AUG-cc-pVQZ method amounts to −3.255 kcal/mol. This value is almost the same as the recent spectroscopic term [126,127] of −3.159 ± 0.029 kcal/mol.

The M06-2X/6–31++G** energy gain in the water-dimer encapsulation inside the $D_{2}$(22)-C_84_ cage is calculated [37,42] as −17.4 kcal/mol, while for the $D_{2d}$(23)-C_84_ cage, it is −14.4 kcal/mol. The terms include the BSSE correction and even the so-called steric correction [56]. The energy gain from various approaches [37] is consistently larger for the (H_2_O)_2_@$D_{2}$(22)-C_84_ encapsulation. The calculated data [37] lead to the finding that encapsulation of (H_2_O)_2_ into both studied C_84_ cages should be connected with a significant energy gain. This finding (when considered with the calculated increase [37,128] of the water-dimer population in the saturated water vapor with temperature) indicates the C_84_ isomers as further potential targets for the experimental investigation of fullerenes with the encapsulated water dimers.

The above-mentioned steric correction [56] basically reflects the cage distortion. First, the complete geometry optimization of the complex species (H_2_O)_2_@C_84_ is performed for both (H_2_O)_2_@$D_{2}$(22)-C_84_ and (H_2_O)_2_@$D_{2d}$(23)-C_84_ endohedrals. Traditionally, the BSSE treatment deals with the geometry of the components straightforwardly taken over from the already optimized complex aggregate. However, the steric-corrected BSSE treatment [56] deals with the corresponding optimized empty C_84_ cage. Such steric correction is also evaluated for the water dimer. Interestingly, the computed O-O distances (2.745 and 2.680 Å in the $D_{2}$ and $D_{2d}$ cage, respectively) are somewhat shorter compared to the observed value [129,130] of 2.98 ± 0.04 Å in the free (H_2_O)_2_.

The encapsulation equilibrium constants for processes (8) and (9) can also be evaluated [39]. The thermodynamic treatment [39] shows that the population ratio of (H_2_O)_2_@$D_{2}$(22)-C_84_ and (H_2_O)_2_@$D_{2d}$(23)-C_84_ decreases with increasing temperature, approaching 2:1 for elevated temperatures.

Finally, simultaneous encapsulations of H_2_O and (H_2_O)_2_ in the $D_{2}$(22)-C_84_ cage are calculated in ref. [38]. In the M06-2X/6–31++G** approach, the monomer encapsulation in $D_{2}$(22)-C_84_ provides a gain in energy of about −10.7 kcal/mol. The ratio between (H_2_O)_2_@C_84_ and H_2_O@C_84_ is also evaluated [38] using the encapsulation equilibrium constants and is close to 1:2.

Similar stability calculations can also be carried out [42] for (H_2_O)_3_ encapsulation in the $D_{2}$(22)-C_84_ fullerene cage:

(10) $${\left(H_{2}O\right)}_{3}\left(g\right)+D_{2}\left(22\right)-C_{84}\left(g\right)={\left(H_{2}O\right)}_{3}@D_{2}\left(22\right)-C_{84}\left(g\right).$$

**Figure 3:**
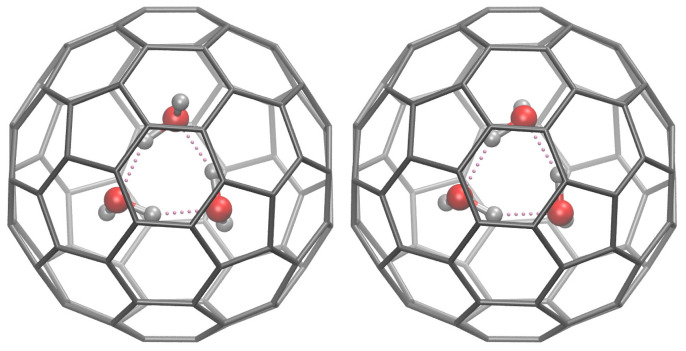
The M06-2X/6–31++G**-optimized structures [42] of (H_2_O)_3_@$D_{2}$(22)-C_18_: left—trans-organization of H atoms not involved in the hydrogen bonds ($C_{1}$ species); right—cis-organization ($C_{3}$ species).

**Figure 4:**
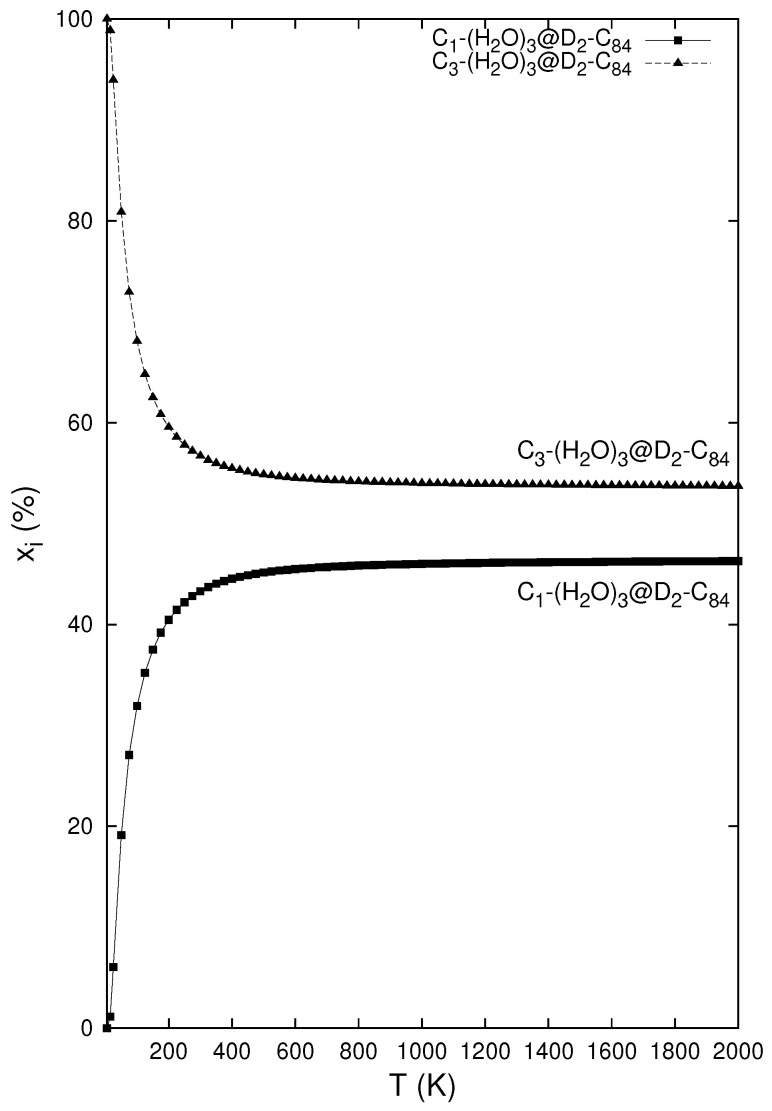
Relative concentrations [42] of the (H_2_O)_3_@$D_{2}$(22)-C_84_ isomers computed at the M06-2X/6–31++G** level.

For example, if the energy gain is calculated for the cyclic (H_2_O)_3_ encapsulation in $D_{2}$(22)-C_84_ using the M06-2X/6–31++G** approach including the BSSE error, it is concluded that the water trimer encapsulation in C_84_ leads to the gain in the potential energy of −10.4 kcal/mol. The encapsulated water trimer can exhibit two different organizations: either the structure present in the free gas-phase water trimer (*trans,* $C_{1}$ point group of symmetry) or the conformation in which three H atoms (not involved in the H-bond) are on the same side of the O-O-O plane (*cis,* $C_{3}$ point group of symmetry) (Figure 3). The species differ in energy by only 0.071 kcal/mol. Their gas-phase equilibrium

(11) $$C_{3}-{\left(H_{2}O\right)}_{3}@D_{2}\left(22\right)-C_{84}\left(g\right)=C_{1}-{\left(H_{2}O\right)}_{3}@D_{2}\left(22\right)-C_{84}\left(g\right)$$

should yield comparable concentrations at higher temperatures. The isomeric concentrations can be calculated [131,132] with the M06-2X/6–31++G** structure and vibrational and energy parameters for the construction of the partition functions (in the commonly used rigid rotor and harmonic oscillator approximation). As can be seen [42] in Figure 4, the concentrations become nearly equimolar quite quickly. At room temperature, the species $C_{3}-{\left(H_{2}O\right)}_{3}@D_{2}\left(22\right)-C_{84}$ (with lower potential energy) represents 57% of the equilibrium mixture.

## 8. Encapsulation of H_2_O and HF in C_70_

HF and H_2_O can be encapsulated in the C_70_ IPR fullerene cage leading to the observed [41] H_2_O·HF@C_70_ species:

(12) $$H_{2}O\left(g\right)+\mathrm{HF}\left(g\right)+C_{70}\left(g\right)=H_{2}O\cdot \mathrm{HF}@C_{70}\left(g\right).$$

The encapsulation energy for process (12) calculated using the B2PLYPD/6–31++G** method with the CP3 (case of three reaction components) BSSE correction (including the steric term) is equal [45] to −25.8 kcal/mol (when applying the 6–311++G** atomic basis to −26.0 kcal/mol). The encapsulation energy is evaluated in the M06-2X/6–31++G**-optimized structures. In fact, the M06-2X/6–31++G**-calculated structure [45] of the H_2_O·HF@C_70_ species agrees reasonably well with the observed characteristics [41]. In particular, the observed hydrogen bond is 1.39 Å long, while its calculated length is 1.481 Å, and the experimental F-O distance of 2.438 Å is also close to the calculated distance of 2.447 Å. It should be noted that the computations deal with a free (gas-phase) H_2_O·HF@C_70_, while the X-ray observation [41] deals with a porphyrin co-crystal. Moreover, the observed [133] dissociation energy for the free H_2_O·HF dimer itself is also well reproduced [45] with the B2PLYPD/6–311++G** method. The obtained equilibrium constant for encapsulation [45] corresponds to the terms calculated [44] for other studied encapsulations. Hence, it is not excluded that H_2_O·HF@C_70_ could also be produced by the direct catalytic high-pressure and high-temperature synthesis [2,13].

## 9. Conclusions

The surveyed cases document that the computational evaluations can fruitfully cooperate with observations of the fullerene endohedrals containing non-metallic encapsulates and thus support and rationalize the experimental findings. The results therefore encourage further such computational studies of nanocarbon endohedrals with encapsulated non-metal species [44,93,134,135,136,137,138,139,140,141,142,143,144,145,146,147,148,149,150] such as H_2_, N_2_, CO, HF, NH_3_, H_2_O_2_, H_2_O, and their aggregates. Future studies should further develop predictions of stability and even populations by applying still more advanced quantum-chemical approaches. Moreover, partition functions for the description of temperature effects should also be further refined so that the description can be based not only on the encapsulation potential energy changes but increasingly also on the encapsulation Gibbs energy terms.

## Figures and Tables

**Table 1:** Illustrative examples of the calculated encapsulation energies.

Encapsulation Process	Encapsulation Energy [kcal/mol]	Ref.
$H_{2}\left(g\right)+C_{60}\left(g\right)=H_{2}@C_{60}\left(g\right)$	−4.2	[30]
$N_{2}\left(g\right)+C_{60}\left(g\right)=N_{2}@C_{60}\left(g\right)$	−9.3	[26]
$\mathrm{CO}\left(g\right)+C_{60}\left(g\right)=\mathrm{CO}@C_{60}\left(g\right)$	−12.5	[40]
${\mathrm{NH}}_{3}\left(g\right)+C_{60}\left(g\right)={\mathrm{NH}}_{3}@C_{60}\left(g\right)$	−5.2	[26]
$H_{2}O_{2}\left(g\right)+C_{60}\left(g\right)=H_{2}O_{2}@C_{60}\left(g\right)$	−12.4	[43]
${\mathrm{CH}}_{4}\left(g\right)+C_{60}\left(g\right)={\mathrm{CH}}_{4}@C_{60}\left(g\right)$	−13.9	[46]
${\left(H_{2}O\right)}_{2}\left(g\right)+C_{70}\left(g\right)={\left(H_{2}O\right)}_{2}@C_{70}\left(g\right)$	−18.4	[42]
${\left(H_{2}O\right)}_{2}\left(g\right)+C_{84}\left(g\right)={\left(H_{2}O\right)}_{2}@C_{84}\left(g\right)$	−17.4	[42] ^*a*^
${\left(H_{2}O\right)}_{3}\left(g\right)+C_{84}\left(g\right)={\left(H_{2}O\right)}_{3}@C_{84}\left(g\right)$	−10.4	[42] ^*a*^
$H_{2}O\left(g\right)+\mathrm{HF}\left(g\right)+C_{70}\left(g\right)=H_{2}O\cdot \mathrm{HF}@C_{70}\left(g\right)$	−26.0	[45]

^a^ $D_{2}\left(22\right)\text{-}C_{84}$.
